# An update of the effects of vitamins D and C in critical illness

**DOI:** 10.3389/fmed.2022.1083760

**Published:** 2023-01-11

**Authors:** Aileen Hill, Christina Starchl, Ellen Dresen, Christian Stoppe, Karin Amrein

**Affiliations:** ^1^Department of Anesthesiology, University Hospital RWTH Aachen, Aachen, Germany; ^2^Department of Intensive and Intermediate Care, University Hospital RWTH Aachen, Aachen, Germany; ^3^Klinische Abteilung für Endokrinologie und Diabetologie, Klinik für Innere Medizin, Medizinische Universität Graz, Graz, Austria; ^4^Department of Anaesthesiology, Intensive Care, Emergency, and Pain Medicine, University Hospital Würzburg, Würzburg, Germany; ^5^Department of Cardiac Anesthesiology and Intensive Care Medicine, Charité-Universitätsmedizin Berlin, Berlin, Germany

**Keywords:** critical care, vitamin D, vitamin C, medical nutrition therapy, micronutrients, review

## Abstract

Many critically ill patients are vitamin D and vitamin C deficient and the current international guidelines state that hypovitaminoses should be compensated. However, uncertainty about optimal dosage, timing and indication exists in clinical routine, mainly due to the conflicting evidence. This narrative review discusses both micronutrients with regards to pathophysiology, clinical evidence of benefits, potential risks, and guideline recommendations. Evidence generated from the most recent clinical trials are summarized and discussed. In addition, pragmatic tips for the application of these vitamins in the clinical routine are given. The supplementations of vitamin D and C represent cost-effective and simple interventions with excellent safety profiles. Regarding vitamin D, critically ill individuals require a loading dose to improve 25(OH)D levels within a few days, followed by a daily or weekly maintenance dose, usually higher doses than healthy individuals are needed. For vitamin C, dosages of 100–200 mg/d are recommended for patients receiving parenteral nutrition, but needs may be as high as 2–3 g/d in acutely ill patients.

## 1. Introduction

The Medical Nutrition Therapy (MNT) is an integral part of the complex care needed for patients in the intensive care unit (ICU). It is especially important for patients with high nutritional risk, for example patients who are chronically malnourished or sarcopenic, or for patients remaining on the ICU for a prolonged period. MNT encompasses the administration of macronutrients (carbohydrates, protein, and fats), as well as the supplementation of micronutrients, for example vitamins and trace elements.

The MNT in the ICU is anything but trivial, the optimal administration strategy frequently remains controversial due to inconclusive evidence. This is especially true for micronutrients, as many of them have been studied in heterogenous ICU populations in varying dosages, routes, and durations of administration at different perioperative and/or ICU times. In addition, reported outcomes differ from trial to trial, preventing rigorous evidence synthesis, for example in forms of well-conducted meta-analyses. Therefore, uncertainty remains about what needs to be considered and to be implemented in the daily clinical practice on ICU.

This article reviews current evidence and controversies about two very different micronutrients—vitamin D and vitamin C—in a narrative manner. Both vitamins are discussed with regards to pathophysiology, clinical evidence of benefits, potential risks, and guideline recommendations. Evidence generated from the most recent clinical trials are summarized. In addition, pragmatic tips for the application of these vitamins in the clinical routine are given.

## 2. Vitamin D

### 2.1. Supplementation with vitamin D in the ICU

Contrary to popular believe, vitamin D is not a vitamin but a steroid hormone with a wide range of cellular, anti-inflammatory, and immunomodulatory effects. It is one of the main regulators of the calcium and phosphate balance, and hence, plays a key role in maintaining a healthy bone metabolism. Moreover, the immunomodulatory features of this hormone are of particular interest for the critically ill patient.

The body obtains vitamin D either from fungi-based diet (vitamin D2) or animal source (vitamin D3), by endogen synthesis in sun-exposed skin (vitamin D3) or by supplements. After that, the liver metabolizes either of them to 25(OH)D, which is further hydroxylated to the active form 1,25(OH)2D in the kidneys. The active form of vitamin D has a half-life of about 1 h and exerts its manifold effects by binding to vitamin D-receptor (VDR), which is expressed by many tissues (1–3) (Table 1).

**TABLE 1:** Overview of available vitamin D preparations, adapted to Prietl B. et al. (4) indicated usual dosage recommendations apply to non-ICU patients.

	Daily dose	Indication	Overdose side effects	Half-life	Costs
**Native vitamin D**					
Unhydroxylated, inactive vitamin D_3_ Cholecalciferol Calcidiol/Calciol Very stable	400–4,000 IU	Vitamin D deficiency, osteoporosis, hypoparathyroidism, rickets prophylaxis.	Rarely hypercalcemia, hypercalciuria	Circulating: 2 days functional: 2–3 months	€
Unhydroxylated, inactive vitamin D_2_ Ergocalciferol Vitamin D2 Less stable during storage, cooking/baking	400–4,000 IU			Circulating: 2 days functional: 2 months or less (33)	€
Calcifediol	30–60 μg	Hyperparathyroidism, Vit D deficiency, renal failure CKD 3–4	Hypercalcemia, hypercalciuria	∼2 weeks	
**Active vitamin D**					
Hydroxylated active vitamin D 1,25(OH)_2_ D_3_ (calcitriol) 1,α(OH)D_3_ (alfacalcidol)	0.25–1.0 μg	Advanced kidney disease with secondary hyperparathyroidism, (pseudo) hypoparathyroidism, not alone in vitamin D deficiency.	Narrower therapeutic range: hypercalcemia, hyperphosphatemia, hypercalcuria, nephrocalcinosis.	15 h	€€
Other active vitamin D analogs: Analogs to vitamin D_2_: Paricalcitol, doxercalciferol Analogs to vitamin D_3_: Falecalcitriol, maxacalcitol		Advanced renal disease with secondary hyperparathyroidism,	Hypercalcemia possible, but less common than with other active forms	∼15 h	€€€

Calcidiol is the storage form of vitamin D from which calcitriol is produced on demand and has the highest half-life of 3 weeks. CKD, chronic kidney disease.

Vitamin D and its metabolites are essential immunomodulators that is involved in many biological processes of the innate and adaptive immune system (4). Immune cells such as lymphocytes, monocytes, macrophages, and dendritic cells express the VDR, which explains the major role of sufficient vitamin D levels for infections and cancer, respectively (5–7). Another key action of vitamin D3 involves its potential to promote the killing of intracellular bacteria such as *Mycobacteria tuberculosis* and M. leprae in macrophages *via* toll-like-receptor 1 and 2, implicating a great capability for severely ill patients (8, 9).

**FIGURE 1:**
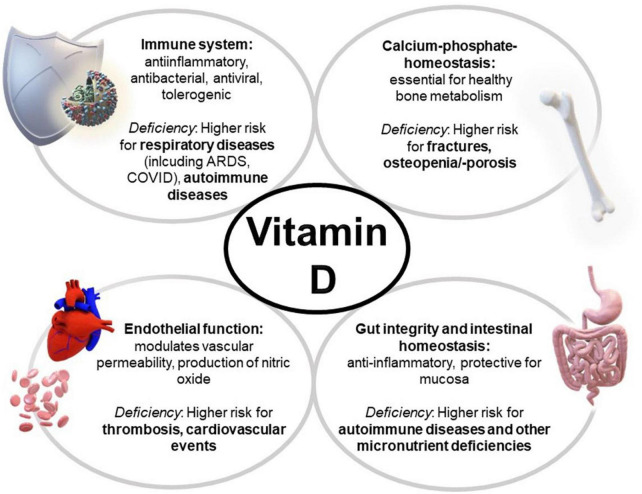
Multiple effects of vitamin D and its deficiency on the body. ARDS, acute respiratory distress syndrome.

Additionally, the VITAL study revealed that daily supplementation with 2,000 IU vitamin D over a course of 5 years appeared to reduce the incidence of autoimmune diseases (10), which could be explained by tolerogenic effects of the hormone (4). Further, vitamin D exerts a protective impact on intestinal mucosa and influences thyroid diseases (6, 11) (see overview—Figure 1).

Vitamin D3 acts *via* both genomic and non-genomic pathways. Basically, calcitriol is thought to exert its effects through interaction with the nuclear vitamin D receptor (nVDR). But there are also non-genomic actions taking place *via* the activation of signaling molecules for example phospholipases C and A2, second messengers, activation of protein kinases or opening calcium or chloride channels. Another non-genomic action includes the binding to target proteins, which provides the basis for the immunoregulatory effects of vitamin D (12).

The actions of vitamin D in a neuronal cell line and the neuroprotective non-genomic effects of vitamin D and its metabolites is still investigated. Recent studies have shown a modulation of synaptic transmission in juvenile GnRH neurons by vitamin D3 and additionally, calcipotriol (a vitamin D analog) hast been shown to act as a neuroprotective agent in neuroblastoma cells (13, 14).

Furthermore, pharmacogenomics have a relevant impact on the pharmacokinetics and -dynamics of vitamins and influence the effectiveness of any supplementation. Vitamin D production strongly depends on renal 1a-hydroxylase, which can be up-regulated by parathyroid hormone (PTH) and inhibited by calcitriol itself. Genetic polymorphisms in the encoding enzymes CYP27B1 as well as CYP24A1 have shown to influence significantly the concentration of vitamin D metabolites in circulation (15).

Also, genetic aberrations in the vitamin D binding protein (VDBP) have shown to be associated with a higher risk for Graves’ disease (16) and have indicated a decreased risk for osteoporosis (17).

Other genetic polymorphisms in diverse CYP-enzymes can lead to remarkably reduced conversion to calcitriol, and genetic variations in the vitamin D receptor have been linked to increased incident of hip fracture, myocardial infarction, cancer and mortality overall. There is some indication that VDR polymorphisms modulate especially the risk for prostate, breast, colorectal and skin cancer (15, 18, 19).

### 2.2. Vitamin D deficiency

Vitamin D deficiency [defined as 25(OH)D levels below 20 ng/ml] is common and occurs in nearly half of the normal population and in about 70% of all intensive care patients (20, 21). Burn patients are particularly affected (22, 23). Vitamin D deficiency can be caused by low seasonal sun exposure or high air pollution (1), vegan diet (24), using sunscreen, staying indoors, wearing concealing clothing and reduced endogenous vitamin D production efficiency from solar UVB in older age.

Also, the bioavailability of vitamin D decreases in patients with obesity (25). In addition, obesity entails systemic inflammation, which may increase the need for exogenous vitamin D.

However, vitamin D deficiency seems to occur in ICU patients worldwide regardless of latitude, i.e., UV exposure (26). Pre-existing conditions, malnutrition, liver, renal or parathyroid dysfunction can lead to reduced vitamin D levels, as can therapeutic interventions and co-medications. All in all, this can result in increased mortality and poorer outcomes (1, 27).

A clear association between low vitamin D and poor clinical outcome in critically ill adults and children has been demonstrated repeatedly in observational studies. There is a higher risk for sepsis, acute respiratory failure, acute renal failure, prolonged ICU stay, as well as increased mortality and morbidity for patients with vitamin D deficiency (21, 28–32).

### 2.3. Previous RCTs in critical care/population settings and methodological issues

In 2014, the VITDAL study showed no benefit of vitamin D supplementation on the primary endpoint in 480 mixed critically ill patients with vitamin D deficiency (defined as 25OHD < 20 ng/ml), using a large upfront loading bolus of 540,000 IU followed by monthly maintenance doses of 90,000 IU. However, in the predefined subgroup with severe vitamin D deficiency (<12 ng/ml), 28-day mortality was significantly lower in the vitamin D group (34).

Several smaller trials followed, using different intervention protocols (oral, intramuscular, and single bolus), showing inconsistent results. The largest published RCT to date (>1,000 patients), the VIOLET study (35), showed no benefit of vitamin D in a less severely ill patient group (patients “with risk for ARDS” were included outside of the ICU, i.e., in the theater, the ward, or the emergency room). The intervention was a single large bolus dose of 540,000 IU vitamin D3. Currently, the European VITDALIZE study (sample size target = 2,400 patients) is still recruiting in several countries including Austria and the United Kingdom using a large bolus dose followed by daily 4,000 IU in a severely deficient ICU population. A direct comparison of the VIOLET study (35) and VITDALIZE study is presented in Table 2.

**TABLE 2:** Comparison of the large intervention studies VIOLET and VITDALIZE—two studies that at first glance appear similar, but nevertheless have significant differences, regarding drug administration, inclusion criteria, and primary endpoints.

	VIOLET (NCT03096314)	VITDALIZE (NCT03188796)
Inclusion criteria	Vitamin D level ≤ 20 ng/ml	25(OH)D ≤ 12 ng/ml
Number of cases	Planned 3,000, aborted after 1,360 at first interim analysis due to “futility.” Analyzed 1,078	Planned 2,400, an interim analysis As of October 2022: 900 patients included
Population	Patients at risk for ARDS	ICU-patients
Bolus output	A single bolus of 540,000 IU	Bolus administration of 540,000 IU and additional daily administrations of 4,000 IU over a 90-day period (corresponding to a total vitamin D administration of 900,000 IU over a 90-day period).
Primary endpoint	90-day mortality	All-cause mortality after 28 days

In the VIOLET study, 1,360 patients were screened and found to have vitamin D levels of <20 ng/ml using point of care testing, but only 1,078 who actually had a vitamin D level <20 ng/ml in subsequent LC/MS testing were analyzed. However, in the previous VITDAL-ICU study, only the subgroup with levels <12 ng/ml showed significantly reduced 28-day mortality, not the subgroup with vitamin D between 12 and 20 ng/ml (34). Based on these results, the design of the VITDALIZE study was planned and created (correct subgroup, correct endpoint). Mention should also be made of the VITDALIZE-Kids study (NCT03742505), led by JD McNally in Canada, which has included critically ill children since 2019 (planned 766, as of October 2022: >200; primary endpoint: health related quality of life).

In a meta-analysis published in 2022, Menger et al. found meaningful and significant benefits of vitamin D administration in critically ill patients for mortality (risk ratio 0.78), shorter ICU stay (–3.1 days), and shorter duration of ventilation (–5.1 days). The aggregated evidence further highlighted the importance of the different vitamin D metabolites and the route of administration (36).

Generally, clinical trials often showed no treatment benefit of vitamin D. Many previous intervention studies displayed multiple, fundamental methodological errors, including e.g., investigation of a non-vitamin D deficient study population, very small case numbers, exaggerated/unrealistic effect sizes, very different specifications on dose and metabolites used (see overview Table 1), or an intake of vitamin D in the placebo group. Previous vitamin D studies were virtually always “underpowered” for the intended endpoint.

In 2014 Heaney published his guidelines with a system to standardize clinical studies of nutrient effects, taking into account the physiology of the investigated nutrient and necessary basics for nutrient study design (37).

For example, in the U.S. VITAL Study with a relatively healthy study population of 25,871 subjects who received vitamin D supplementation of 2,000 IU for 5 years with or without omega-3 fatty acids, neither of which demonstrated protection against falls, cardiovascular events, or cancer (38, 39). There were many problems with the design and analyses of the study, including that vitamin D status before the start of supplementation was normal and averaged 30.8 ng/ml, whereas only 12.7% of participants had levels below 20 ng/ml. One explanation could be that vitamin D is often added to American foods such as milk and participants were allowed to consume up to 800 IU (20 μg) of vitamin D daily, which is the usual daily recommendation for adults. Additionally, BMI overall was high and outcomes were not evaluated individually concerning the particular 25OHD concentration.

However, secondary analysis concluded that all-cancer mortality rate was significantly reduced, especially in the cohort with normal weight. Obesity and being overweight seemed to obstruct this effect (40).

Another important variable is adequate sample size, which in randomized trials is primarily determined by the estimated effect of the intervention relative to the standard/placebo group, but also the desired power. For example, a very high number of cases is required to detect an effect on mortality. In the future, it might be helpful to use composite endpoints (primary endpoints consisting of several individual endpoints)—as it has been common practice in other fields, including critical care and cardiology, for a long time (41–43).

### 2.4. High dose bolus administration

By now, one-time high-dose supplementation is considered obsolete, as it has been shown to be ineffective, and in some settings even harmful (in contrast to a daily/weekly administered vitamin D dose). Not even the exemplary vitamin D deficiency disease—rickets—can effectively be treated with bolus administration (44). Biologically, this can probably be explained by the induced activation of catabolic enzymes 24-hydroxylase and FGF-23, both of which can inactivate vitamin D (45). Likewise, in the prevention of respiratory infections only daily or weekly low-dose administration is effective (46). Nevertheless, in the intensive care setting—since it is a time-critical situation—a high-dose loading dose could be given at the beginning, followed by maintenance therapy (47).

### 2.5. Implications for COVID-19

Patients with low vitamin D levels are at increased risk of becoming infected with SARS-CoV-2 and developing severe disease including ICU admission and death (48). A recent study by Gibbons et al. has shown that the supplementation of vitamin D2 and D3 reduced the risk for COVID-19 infection, as well as mortality. The benefits were even better in people receiving higher dosages, as well as in those with a more severe deficiency (49).

Vitamin D appears to have multiple effects on cells and tissues that have also been implicated in the progression of COVID-19 and of ARDS. Vitamin D is a regulator of important genomic and non-genomic mechanisms in the immune system, is involved in the defense against respiratory infections, and also plays a controlling role in the renin-angiotensin-aldosterone system used by SARS-CoV-2 to invade host cells (50). Epithelial cells are the primary target of respiratory pathogens, and a seasonal decline of vitamin D could contribute to a higher rate of (lower) respiratory tract infections during winter. Two meta-analyses showed that vitamin D treatment can reduce the risk for respiratory tract infections (5, 46). Respiratory epithelial cells, as well as macrophages and monocytes express enzymes in response to inflammation, which are able to convert 25(OH)D into the active form 1,25(OH)2D25, which in turn induces the expression of the antibacterial and antiviral peptide cathelicidin and influences lymphocyte activity (51). 1,25(OH)2D affects immunotolerance in antigen-presenting cells (APC) (52); hence, having a tolerogenic impact, by decreasing IL-12 production and increasing IL-10 (53).

Vitamin D-receptors are also expressed by blood vessels, whereby vitamin D modulates endothelial function as well as vascular permeability, and it is a transcriptional factor for endothelial nitric oxide production (54). Lack of vitamin D has been associated with thrombotic and cardiovascular events and has shown *in vitro* to influence the structure and formation of thrombi. In severe vitamin D deficiency, a high-dose cholecalciferol supplementation was associated with a reduction in thrombin generation (55, 56). This potential impact may play a role in the future in the prevention and treatment of COVID-19 patients, which often suffer from coagulopathy, thrombosis and microangiopathy.

A small RCT including 50 patients to evaluate high-dose calcifediol administration compared to standard therapy without calcifediol has shown to cause a significant reduction in the need for ICU treatment of hospitalized patients (57). Calcifediol is a more rapidly acting native vitamin D metabolite that is currently only available for routine purposes in some countries but is certainly interesting in the ICU population. Oral calcifediol increases the serum 25(OH)D levels more rapidly and is about 3.2-fold more potent than cholecalciferol, which means that lower dosages are needed. Also, intermittent oral calcifediol showed a higher rate of intestinal absorption and potentially a more stable serum 25(OH)D level than intermittent oral cholecalciferol intake (58).

A systematic review and meta-analysis including 2,078 COVID-19 patients found that 61 (10.5%) individuals in the vitamin D supplementation group died, compared to 386 (25.8%) in the non-treated group (OR: 0.60; 95% CI: 0.32–1.12; *p* = 0.11). Regarding ICU admission, 12.2% of the individuals in the treated group were admitted to ICU, compared to 26.3% in the non-treated group (OR: 0.33; 95% CI: 0.15–0.71; *p* = 0.005) (59).

Considering the multiple effects that vitamin D exerts throughout the human body and the data we have so far on the topic, the achievement of an adequate level of vitamin D could play a highly relevant role as an adjuvant tool to optimize immunologic responses to infection or vaccination. However, it may not be possible to show a small benefit during the pandemic because of the high financial and regulatory barriers and the time needed to collect such data.

### 2.6. Fracture risk and osteoporosis after critical illness

The risk of fracture after an ICU-stay is increased (60). Accelerated bone resorption, including changed bone turnover markers, bone mineral density and fragility fracture rate are issues in critically ill patients and predict mortality. The reasons include immobilization and resulting muscle loss, frequent vitamin D deficiency, inflammatory cytokines, but also malnutrition, as well as drugs such as steroids or proton pump inhibitors (PPI) (61, 62).

Early mobilization and restrained medication with PPIs and steroids are cornerstones of osteoporosis prophylaxis in this setting. Interestingly, bisphosphonates have been shown to significantly improve survival in patients at particularly high risk of mortality (63). In cases of prolonged immobilization, antiresorptive substances such as bisphosphonates or denosumab could be tried as a case-by-case decision (64). The basic prerequisite for this is an adequate vitamin D level as well as sufficient calcium intake, otherwise severe hypocalcemia may occur. The results of the currently recruiting Australian Bone Zone study will be highly relevant (NCT04608630) for the question if zoledronic acid or denosumab can improve bone health in this special setting.

### 2.7. Target levels

The target vitamin D level indicating optimal levels remains highly controversial and different targets may apply to different diseases. A high dose supplementation to reach pharmacological levels could be required to achieve some effects, but the potential for toxicity must be kept in mind when using such an approach.

### 2.8. Key messages (vitamin D)

• In case of (par)enteral nutrition, vitamins and trace elements should always be supplemented.
• Usual daily requirement for adults: 600–4,000 IU of vitamin D daily.
• Vitamin D deficiency is a rapidly modifiable risk factor in critically ill patients.
• The 25(OH)D level is a relatively stable and now readily available laboratory value after the hyperacute phase of acute illness/outside the initial phase of major surgery that reliably detects vitamin D deficiency (<20 ng/ml).
• Given the available evidence, we suggest a target level of 30–40 ng/ml and a repeat 25OHD measurement after a few weeks.
• Vitamin D deficiency has been described in the majority of ICU patients worldwide (children and adults).
• Vitamin D supplementation thus represents a cost-effective, simple intervention with an excellent safety profile that is involved in multiple biological processes of the immune system, bone health, and mortality, and thus could contribute significantly to improved outcomes in intensive care patients.
• Critically ill individuals may require a loading dose to improve 25(OH)D levels within a few days, followed by a daily or weekly maintenance dose, usually higher doses than healthy individuals are needed.
• For a reduction of the increased fracture risk after critical illness, antiresorptive substances such as bisphosphonates/denosumab should be considered as a case-by-case decision in prolonged immobilization; the basic prerequisite for this is an adequate vitamin D level as well as sufficient calcium intake, otherwise severe hypocalcemia may occur as an adverse event.
• In the future, calcifediol may play a more important role in the ICU setting, as it seems to increase 25(OH)D serum concentrations faster than oral vitamin D3.
• Side effects of vitamin D supplementation are rare and include:
  ○ Hypercalciuria/-calcemia.
  ○ Kidney damage and new formation of kidney stones.
  ○ Increased risk of falls and fractures has been described for infrequent or single ultrahigh doses.

## 3. Vitamin C

Vitamin C is the first recognized and first synthetically fabricated vitamin. The pharmacokinetics and metabolic pathways have been discussed extensively in several reviews (65, 66), therefore only a short summary is given here. Vitamin C is a water-soluble, essential micronutrient with a multitude of functions in the human body. Vitamin C has almost complete bioavailability after enteral resorption and is eliminated *via* the kidneys. In the plasma, it exists in two forms: ascorbic acid and its oxidized and biologically less active form dehydroascorbate. Vitamin C is quickly eliminated with an approximate half-life of 1 h after intravenous injection. Vitamin C is required for the function of more than 60 enzymes, among which are (65):

• The synthesis of norepinephrine, collagen, and carnitine.
• The function of vitamin C dependent mono- and dioxygenases involved in peptide amidation and tyrosine metabolism.
• The metabolism of cholesterol to bile acids and in steroid metabolism.
• The cytochrome P450 driven hydroxylation or aromatic drugs and carcinogens.
• The absorption of iron in the small intestine.
• Stem cell differentiation.

**FIGURE 2:**
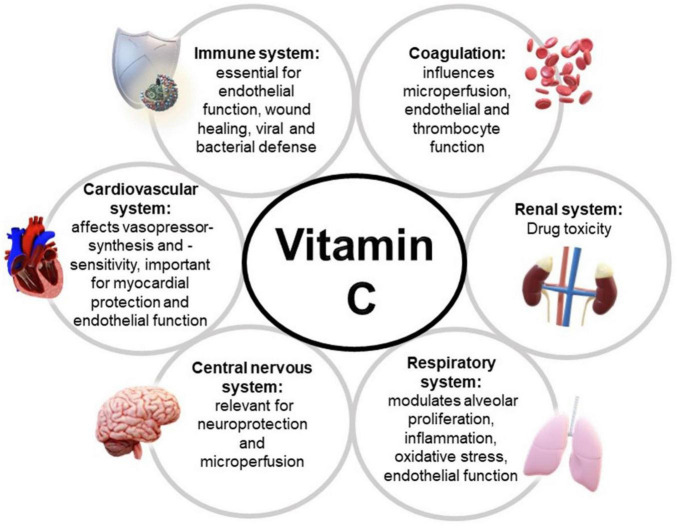
Vitamin C’s influence of different organ systems.

Vitamin C is also a strong antioxidant and counterbalances the influence of reactive oxygen species thus protecting cells and organs from damage in settings of oxidative stress and inflammation (65). A very condensed overwiev of vitamin C’s inflence on different organ systems is displayed in Figure 2. Within the specific contents of this manuscript, it is important to mention that vitamin C raises glutathione levels and may thereby increase the effect of vitamin D as well (67–71).

In the western-countries, patients with the full clinical symptoms of scurvy (gingivitis, hemorrhagic diathesis, immune deficiency pancytopenia, general weakness) are not to be expected frequently due to overall good supply situation (72). Nevertheless, in critical illness there are many different patient populations who may benefit from vitamin C supplementation, especially since ICU patients regularly show suboptimal vitamin C levels (73–75), which ultimately lead to compromised function of the immune system and antioxidant defense mechanisms.

### 3.1. Potential benefits of vitamin C in critical illness

There are many different ICU subpopulations possibly profiting from vitamin C administration, as vitamin C has pleiotropic functions.

The greatest benefit of vitamin C is to be expected in patients with high extend of inflammation and oxidative stress, as vitamin C not only neutralizes free radicals and reactive oxygen species, but also restores other antioxidative molecules, such as vitamin E. Therefore, vitamin C may reduce cell and organ damage. A typical case is ischemia-and reperfusion injury (I/R-injury). As examples, benefits of a vitamin C supplementation were shown in cardiac surgery with the use of cardio-pulmonary-bypass (76–82), patients after cardio-pulmonary resuscitation or patients with surgeries requiring the clamping of large arterial vessels (65). In experimental models, such as ischemic stroke, vitamin C proved to be beneficial as well (83–85).

Another patient group who may potentially benefit from vitamin C administration, is patients experiencing systemic inflammation, for example after polytrauma, burn injury, or during sepsis and shock. In this patient group, vitamin C is involved in vasopressor synthesis, improves the endothelial sensitivity for vasopressors, as well as endothelial function and microperfusion, which in turn can reduce edema and volume demands (65, 86, 87).

The two mentioned clinical situations—oxidative stress and systemic inflammation—are attributed special attention in the new Micronutrient guidelines of the ESPEN (European Society for Clinical Nutrition and Metabolism) (47). These patients should receive 2–3 g/d vitamin C intravenously, whereas other patients should receive 100–200 mg/d (47). However, it must be acknowledged that these recommendations given are based on the evidence of predominantly smaller RCTs, which are known to overestimate the effects size.

In addition, patients who experience greater losses of water-soluble micronutrients, for example during extracorporeal circulation (e.g., with hemofiltration or extracorporeal membrane oxygenation or patients with significant exudation (large wound surface, thermal injury, open abdomen) may need a greater amount and parenteral administration of vitamin C when compared to other patients (88).

Two other mechanisms come in mind, where vitamin C supplementation could be useful, although here the pathomechanisms are less acute. Patients with chronically high oxidative stress, for example patients with coronary artery diseases or chronic obstructive pulmonary disease also benefited from vitamin C in past clinical trials (65). Furthermore, vitamin C reduced drug-toxicity in several settings, even though this currently finds sparse clinical application. Vitamin C reduced the risk of contrast-mediated nephropathy (89) and reduced drug-toxicity, apoptosis and DNA damage during radio- and/or chemotherapy (90, 91). The following table (Table 3) gives a condensed summary of the pathologies, pathomechanisms and the described benefits of vitamin C on different patient cohorts. It must be pointed out that this table does not discriminate the level of evidence between individual sources. The reason is simple: a rigorous and methodologically meticulous discussion of all high-level evidence regarding the administration of vitamin C across all the mentioned medical fields would be beyond the scope of this review and needs to be done elsewhere.

**TABLE 3:** Role and potential benefits of vitamin C on various organ systems, adapted from; all described benefits were derived from clinical studies unless stated explicitly.

Patient group/Afflicted organ system	Special role of vitamin C in the organ system	Pathology/Condition studied	Pathophysiology related to potential usefulness of vitamin C	Benefits from vitamin C administration						
				Described benefits of vitamin C in the organ system/Condition	Described or potential mechanisms through which vitamin C may be beneficial					
					Mitigation of oxidative stress + restoration of other antioxidants	Improved endothelial function and microcirculation	Improved vasopressor response	Improved platelet function and decreased capillary plugging	Reduced extravasation and edema	Enhanced immune function and antibacterial properties
Central nervous system	● Elevated levels up to 80 times to protect neurons (92, 93) ● Essential for the differentiation and myelinization of neurons (94, 95)	Stroke/Cerebral ischemia	● I/R injury and subsequent oxidative stress ● Shift of vitamin C from the intra- to extracellular compartment and intraneuronal vitamin C deficiency (75)	● Reduces infarct volume in experimental models (83–85) ● Decreases ischemic stroke-related lipid peroxidation (96)	×	×		×	×	
		ICB Head trauma	● Hemorrhage/tissue damage ● Increased ICP and subsequent reduction of cerebral perfusion	● Inversely correlated with major diameter of lesion and severity of neurological impairment (97)	×	×	×	×	×	×
Cardiovascular system	● Frequent vitamin C deficiency (98) ● Vasopressor synthesis	Cardiac surgery Post-reanimation	● I/R Injury and subsequent oxidative stress ● Hemodilution and extracorporeal circulation ● Compromised cardiac function and hemodynamics	● Decreases myocardial injury (99) and higher cardiac index (99) ● Decreases rate of post-op arrhythmia (76–82), ICU (76, 79, 80) and hospital stay (76, 79–81, 100) and ventilation time (76, 77, 81) ● Decreases bleeding (101)	×	×	×	×	×	×
		CAD and MI	● Chronic inflammatory disease leading to increased need of antioxidants	● Promotes endothelial and NO dependent vasodilation ● Reduces AKI after coronary angiography (89)	×	×	×	×		
Respiratory system	● High levels in alveolar type II cells and macrophages (102) ● Collagen synthesis, endothelial restoration and alveolar proliferation (103)	ARDS Pneumonia COPD Asthma Cystic fibrosis	● Most common organ system to suffer from reactive oxygen species (103) ● Acute or chronic inflammation leading to increased need of antioxidants ● Plasma histamine levels correlate inversely with vitamin C levels (102)	● Reduces I/R injury and lung damage in experimental models (65) ● Reduces pulmonary inflammation (104) ● Reduces mortality in ARDS (105) ● Prevents/ameliorates pneumonia (106, 107)	×	×	×		×	×
Renal system	● Renal excretion	Contrast mediated nephropathy	● Drug toxicity	● Decreases risk for AKI (89)	×	×				
		COVID-19 SIRS	● Systemic inflammation and oxidative stress ● Vitamin C can reduce the expression of ACE2, which hinders the entry of the virus into cells, and stabilizes blood pressure (108)	● In experimental models: protects kidneys from injuries caused by external factors, facilitates repair (108)	×	×				
Oncology	● Induces pluripotent stem cell differentiation ● Drug metabolism	Chemo- and radiotherapy	● Reduced uptake due to anorexia and cachexia ● Drug toxicity ● Reduced organ function ● Clotting disorder	● Decreases drug toxicity (90) ● Attenuation of apoptosis and DNA damage (91) ● Decreased intestinal mucosa damage in experimental design (109) ● Increases chemosensitivity	×	×				x
Critical illness	● Frequent vitamin C deficiency (73–75) ● Drug metabolism	Sepsis/septic shock,	● Systemic inflammation ● Imbalance between vitamin C loss/requirements and vitamin C uptake	● Reduces vasopressor requirement (86, 87) ● Reduces mortality (86, 87, 105, 110, 111) and organ failure (87) ● Shortens ICU stay (111)	×	×	×	×	×	×
Burn injury	● Induces pluripotent stem cell differentiation ● Collagen and carnitine synthesis	Severe thermal injury with large% TBSA burned Inhalation trauma	● I/R Injury and subsequent oxidative stress ● Hemodilution ● Large wound surfaces and increased losses ● “After-burn” and disturbed microperfusion	● Reduction of resuscitation volume (112–114) ● Shorter time to wound healing (115) ● Reduces vasopressor requirement (113) ● Increases urine output (113, 116) ● Shorter mechanical ventilation (112) ● Improved hospital survival (117)	×	×	×	×	×	×

ACE II, angiotensin converting enzyme II; ARDS, acute respiratory distress syndrome; CAD, coronary artery disease; COPD, chronic obstructive pulmonary disease; ICP, intracranial pressure; ICU, intensive care unit; ICB, intracranial bleeding; I/R, ischemia- and reperfusion; MI, myocardial infarction; NO, nitric oxide; SIRS, systemic inflammatory response syndrome; TBSA, total body surface area.

### 3.2. Potential harms of vitamin C?

Generally, vitamin C is regarded as safe and easy to use micronutrient. Adverse effects were mostly mentioned in case reports and especially after prolonged high-dosage administration. In patients with some hereditary diseases, vitamin C administration led to adverse effects: it can aggravate iron-overload in patients with hemochromatosis, as vitamin C is a co-factor for iron uptake. In patients with G6DP deficiency (glucose-6-phosphate dehydrogenase), which has a high-varying geographical prevalence, the vitamin may induce hemolysis. Patients with severe chronic kidney disease or history of kidney stones need a close monitoring of the kidney function during high-dose vitamin C administration, as the formation of oxalate kidney stones may be induced (65).

High-dose of intravenous vitamin C may alter the measurements of some handheld blood glucose meters, which may measure false-high blood glucose levels. This needs special attention, as true hypoglycemia may be masked or truly euglycemic patients may receive insulin treatment due to false-high readings (65).

Other side effects of vitamin C, such as flatulence and diarrhea or false-positive readings of tests for occult blood in the stool can be attributed to enteral administration of vitamin C. In theory, vitamin C may act pro-oxidatively, although this has not been proven *in vivo* and it remains discussed whether this potential pro-oxidative character might not be beneficial as well (65, 118).

A systematic review of the harmful effects of high dose vitamin C (median 22.5 g/d) showed diverse rates of adverse events (numerically one higher, one equal and one lower in comparison to the control group in each one RCT). In 2,801 patients, five cases of oxalate nephropathy (0.18%), five cases of hypernatremia (0.18%), three cases of hemolysis in G6DP deficient patients (0.11%), two cases of glucometer error (0.07%), and one case of kidney stones (0.04%) were reported (119). In addition, the latest systematic reviews and meta-analyses have shown no difference in (serious) adverse effects in patients receiving vitamin C in clinical studies (76, 110, 111).

### 3.3. Current evidence for vitamin C

In 2017, a retrospective Study from Marik et al. administering a combination of hydrocortisone, vitamin C and thiamine (“HAT-therapy,” “Marik Cocktail”) to patients with severe sepsis and septic shock demonstrated a significant advantage of survival and reduced rate of organ dysfunction in those patients receiving vitamin C (87). Since then, the interest in vitamin C-research augmented largely. Mostly small studies (which in the majority cannot be discussed here for the sake of conciseness) with diverse dosing and administration regimens in heterogeneous patient populations aimed to demonstrate a treatment effect, whereas the reported outcomes and outcome measurement timepoints were as diverse as the rest of the studies.

As examples, the studies from Fowler et al. showed a reduction of inflammatory markers and organ dysfunction in septic patients (120), as well as a significant reduction of mortality in patients with ARDS (acute respiratory distress syndrome) in a large randomized controlled study, the “CITRIS-ALI”-trial (105) in the respective vitamin C groups. Combination therapies, for example with other antioxidants or vitamins (vitamin E, vitamin A), as well as other drugs (statins, acyl cysteine, omega-3 fatty acids, and ß-blocker) have also been evaluated in clinical studies and have mostly shown neutral or even negative effects of these “cocktails” (65, 121–124). Regarding higher level of evidence, there was an equal flood of meta-analyses, which demonstrated benefits of high-dose vitamin C for example in cardiac surgery patients (76, 78–81, 100, 125) or in the common ICU population (110, 126, 127), some also calculated no clear influence of vitamin C on the individual outcomes.

However, this year, in a large randomized controlled trial, the “LOVIT- trial” from Lamontagne et al., vitamin C monotherapy in septic patients was associated with a significantly higher occurrence of the combined endpoint: “death + persistent organ dysfunction,” while the latter was defined as use of vasopressors, invasive mechanical ventilation, or new renal-replacement therapy at 28 days (128). Although this study with 872 patients led to doubts regarding the potential benefits of vitamin C, it should be mentioned that neither the single components of the composite endpoint nor inflammatory markers, nor 6 months-mortality nor quality of life were different between the groups and that the severity of illness was unevenly balanced, so that a null effect rather a harmful is currently discussed (129). In addition, commencement of vitamin C therapy was delayed (>10 h) and with high variance, limiting the generalizability of the findings.

Currently recruiting randomized controlled trials include trials in patients with ARDS, sepsis COVID-19, cardiac surgery, cardiac arrest and burn patients (NCT04138394). The currently registered 254 studies (www.clinicaltrials.gov, accessed October 3rd, 2022, under the search term “vitamin C”) will show if a vitamin C administration really has beneficial treatment effects in critically ill patients.

### 3.4. Vitamin C in the ICU routine

In summary of the above sections, it can be stated that vitamin C has a broad spectrum of potential benefits with relatively few risks of adverse effects. The administration of vitamin C is neither costly nor is it difficult to administer and is usually quickly eliminated *via* the kidneys in case of overdoses. In patients with G6DP-deficiency, hemochromatosis, history of kidney stones or chronic kidney disease, or if blood sugar cannot be determined with reliable devices, vitamin C should not be used in higher dosages. Pathophysiologically, a loading dose of vitamin C may be beneficial, as the greatest amount of inflammation and oxidative stress is usually the greatest in the most acute setting, however, there is no evidence on this strategy yet. Otherwise, the dosing for different patient populations is summarized in Table 4.

**TABLE 4:** Recommendations for vitamin C dosing according to the ESPEN Micronutrient Guideline (47) and nutrition in the ICU guidelines.

Patient group	Examples	Dosage
Parenteral nutrition (47, 130, 131)	Supplementary or total parenteral nutrition	100–200 mg/d (2)
Patients with chronically elevated demands (47)	COPD, CAD, alcoholism, diabetes mellitus, smokers, heart failure, chronic dialysis	200–500 mg/d (2)
Patients in the acute phase of critical illness (47)	I/R Injury: cardiac surgery, stroke, resuscitation, clamping of large vessels, tourniquet Increased losses: extracorporeal circulation, exsudation Systemic inflammation: sepsis, shock, polytrauma	2–3 g/d (47)

CAD, coronary artery disease; COPD, chronic obstructive pulmonary diseases; I/R: ischemia-reperfusion.

Studies have shown that in the general ICU populations, vitamin C levels are often suboptimal (73–75), suggesting that increased vitamin C supplementation might optimize the body’s function. Nevertheless, especially in the setting of critical illness the interpretation of serum or plasma vitamin C levels needs to be performed cautiously, as they are influenced by hemodilution and re-distribution (98). In addition, the measurement of vitamin C is hindered by its pH-, thermo-, temperature-, and UV-lability. Therefore, the measurement is relatively difficult to perform correctly in clinical routine and meticulous probe processing is required to obtain valid results (98). When measurement of the vitamin C status is needed, EDTA tubes should be used to collect plasma. The probes should be centrifuged the supernatant should be transferred into Eppendorf tubes. Afterward and depending on the standards of the collaborating laboratory, denaturization of proteins and additional centrifugation might be necessary. During the whole process, the probes should be cooled, e.g., stored on crushed ice and protected by light, for example through wrapping the probes with tinfoil. From drawing blood until storage at −80°C, the whole process should be performed in under 60 min (90 min in case of protein denaturization) (98).

There is not enough evidence to answer the question of the optimal administration strategy of vitamin C with regard to indication, dosage, route of administration, duration and monitoring. However, the following key messages give a summary for the use of vitamin C in clinical routine.

### 3.5. Key messages (vitamin C)

• Vitamin C has a central role for multiple enzymatic processes in the human biology and is important for an optimal immune function in the human body.
• Vitamin C has a broad spectrum of potential benefits with relatively low risks of adverse effects. The administration of vitamin C is simple and inexpensive, however it needs to be protected from light and heat to avoid degradation.

• Vitamin C deficiency is frequent among ICU patients and should be compensated to allow optimal enzymatic and immune function (47).
• The measurement of vitamin C in the clinical practice remains cumbersome: mistakes during probe-handling, hemodilution and compartment shifts of vitamin C may contribute to false-low measured plasma levels.
• If vitamin C is measured, probe processing needs to be performed quickly (<90 min), cooled and under light-protection.
• There is currently is evidence for high dosages of vitamin C in the general ICU population and guidelines uniformly recommend against the use of vitamin C in pharmacologic dosages (47, 130, 131).
• Dosage should be as follows (47, 130, 131):
  ○ 100–200 mg/d in patients with parenteral nutrition.
  ○ 200–500 mg/d in patients with chronically elevated vitamin C demand.
  ○ 2–3 g/d in patients in the acute phase of critical illness.
• In case of vitamin C supplementation special attention should be paid to possible adverse effects:
  ○ Artificial hyperglycemia.
  ○ Nephropathy and kidney stones.
  ○ Hemolysis.
• Current evidence:
  ○ The recent LOVIT trial questions the benefits of high-dose vitamin C in patients with sepsis and septic shock.
  ○ Systematic reviews suggest benefits of high-dosed vitamin C if given alone in critically ill patients.
  ○ Currently recruiting trials will show if a high-dose vitamin C supplementation may be of use for distinct subpopulations of critically ill patients.
• Point of care tests, which would allow a rapid measurements of vitamin C levels, or its surrogates would be helpful to identify specific patient populations in need for this treatment and/or assist to evaluate the success of this intervention.

## Author contributions

AH and KA: concept. AH, CSa, CSo, and KA: first draft of the manuscript. CSa and AH: figures. AH, CSa, ED, CSo, and KA: proofreading and correction of content and language. All authors contributed to the article and approved the submitted version.
